# Carbon Monoxide Oxidation Promoted by Surface Polarization Charges in a CuO/Ag Hybrid Catalyst

**DOI:** 10.1038/s41598-020-59531-0

**Published:** 2020-02-13

**Authors:** Xijun Wang, Chuanyi Jia, Edward Sharman, Guozhen Zhang, Xin Li, Jun Jiang

**Affiliations:** 1 Hefei National Laboratory for Physical Sciences at the Microscale, CAS Key Laboratory of Mechanical Behavior and Design of Materials, School of Chemistry and Materials Science, University of Science and Technology of China, Hefei, Anhui 230026 P.R. China; 2 Department of Chemical and Biomolecular Engineering, North Carolina State University, Raleigh, North Carolina 27606 USA; 3 Guizhou Provincial Key Laboratory of Computational Nano-Material Science, Institute of Applied Physics, Guiyang, 550018 China; 4 Department of Neurology, University of California, Irvine, California 92697 USA

**Keywords:** Computational chemistry, Electronic properties and materials

## Abstract

Composite structures have been widely utilized to improve material performance. Here we report a semiconductor-metal hybrid structure (CuO/Ag) for CO oxidation that possesses very promising activity. Our first-principles calculations demonstrate that the significant improvement in this system’s catalytic performance mainly comes from the polarized charge injection that results from the Schottky barrier formed at the CuO/Ag interface due to the work function differential there. Moreover, we propose a synergistic mechanism underlying the recovery process of this catalyst, which could significantly promote the recovery of oxygen vacancy created via the M-vK mechanism. These findings provide a new strategy for designing high performance heterogeneous catalysts.

## Introduction

Heterogeneous catalysts of various reactions such as CO oxidation have attracted widespread attention due to their high efficiency, benign environmental impact, and durability^1–3^. Their high catalytic activity is normally ascribed to the characteristically high charge transfer efficiency between adsorbed gas molecules and the catalyst surface, a quality which is highly dependent on the surface charge density of active sites^4,5^. However, precise control of charge location at the atomic level still remains a challenge^6^. Although tremendous attempts have been made, using methods such as shape-controlling^7^, chemical doping^8^, crystal-plane-controlling^9^, use of an external electric field^10^, *etc*., it still remains a considerable challenge to precisely tailor the surface charge state of these solid materials so as to enhance both activity and stability simultaneously^11,12^. The fundamental problem is the limited understanding of the nature of surface polarization, a barrier which seriously hinders the further practical application of heterogeneous catalysts.

**Figure 1:**
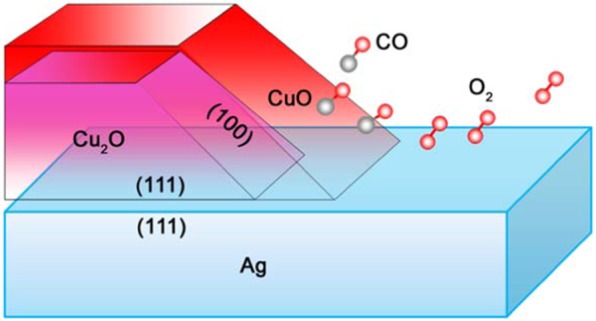
Schematic illustration of the CuO/Ag hybrid structure (supported on Cu_2_O/Ag).

To acquire deeper insight into surface polarization, recently we designed a novel semiconductor-metal interfacial structure (CuO/Ag) for CO oxidation^13^. As shown in Fig. 1, a Cu_2_O(111) surface was first deposited on a flat Ag(111) substrate, tailored to expose a Cu_2_O(100) surface. Following this first treatment cycle, the exposed Cu_2_O(100) surface was then oxidized to form a layer of CuO(100) surface. Compared with previous reported CuO based catalysts^14–18^, our design achieves complete conversion of CO at relatively low temperature (150 °C), which performs better than bare CuO/Cu_2_O^14^ or CuO^15^ (Table 1). Despite the existence of better performing materials^16–18^, our design can still serve as a sample model to study the promotion of charge polarization on catalytic activity. Since the thickness of CuO layer is >10 nm, we only use the CuO/Ag model in our DFT simulations due to the limitation of computational resources. It should be noted that such CuO(100) layers have the same lattice constant with Cu_2_O surface (a = b = 4.27 Å), which is different from normal CuO according to previous reports^14^. Such a unique structure may induce an interfacial polarized charge which would then penetrate the CuO(100) surface owing to the very thin atomic thickness along the interfacial line. Our previous results demonstrated that this polarized charge along the interfacial line enables controllable highly efficient CO oxidation with good durability. By maximizing the Ag/CuO interfacial length, the CO conversion rate can be linearly enhanced.

**Table 1:** CO conversion and reaction temperatures of different CuO based catalysts.

	CO Conversion (%)	Reaction Temperature (°C)
Ag/CuO (ref. ^13^)	100.0	150
CuO/*o*-Cu_2_O (ref. ^14^)	93.2	240
CuO/*c*-Cu_2_O (ref. ^14^)	49.1	240
CuO (ref. ^15^)	22.5	205
CuO/CeO_2_ (ref. ^16^)	99.0	117
Au/CuO_2_ (ref. ^17^)	32.5	88
Au/CuO_x_-TiO_2_ (ref. ^17^)	100.0	80
Au/CuO_2_ in O_2_-rich stream (ref. ^18^)	100.0	55

However, there are still several unsolved problems requiring clarification, such as elucidating the formation process of interfacial polarization, the mechanism of transfer of polarized charge at the interface, and how polarized charge impacts catalyst activity. To gain deeper insight into these inner mechanisms, we now extend our study of this system, so as to provide more confidence and experience in designing new composite functional materials.

## Computational Methods

All the calculations were performed at the spin-polarized density functional (DFT) level of theory as implemented in the Vienna ab initio Simulation package (VASP)^19^. The frozen-core all-electron projector augmented wave (PAW) model^20^ with Perdew-Burke-Ernzerhof (PBE) functionals^21^ was employed to describe the interactions between core and electrons. An energy cutoff of 450 eV was used for the plane-wave expansion of the electronic wave function. The force convergence criterion was set to 0.01 eV/Å for pure Ag and Cu_2_O/CuO unit structures and 0.05 eV/Å for the composite CuO/Ag model, respectively. The energy convergence criterion was set to 10^−5^ eV. A k-point grid with a 7 × 7 × 1 Monkhorst–Pack mesh for pure Cu_2_O/CuO and Ag (100) and (111) facets was chosen for sampling the Brillouin zone, while for the CuO/Ag structure, due to the huge size of the model, only Gamma point was performed. A Bader analysis of charge density of atoms was used to analyze the charge distribution^22^, and the nudged elastic band (NEB) method^23^ was employed to search the transition states of elementary steps during the CO oxidation reaction.

**Figure 2:**
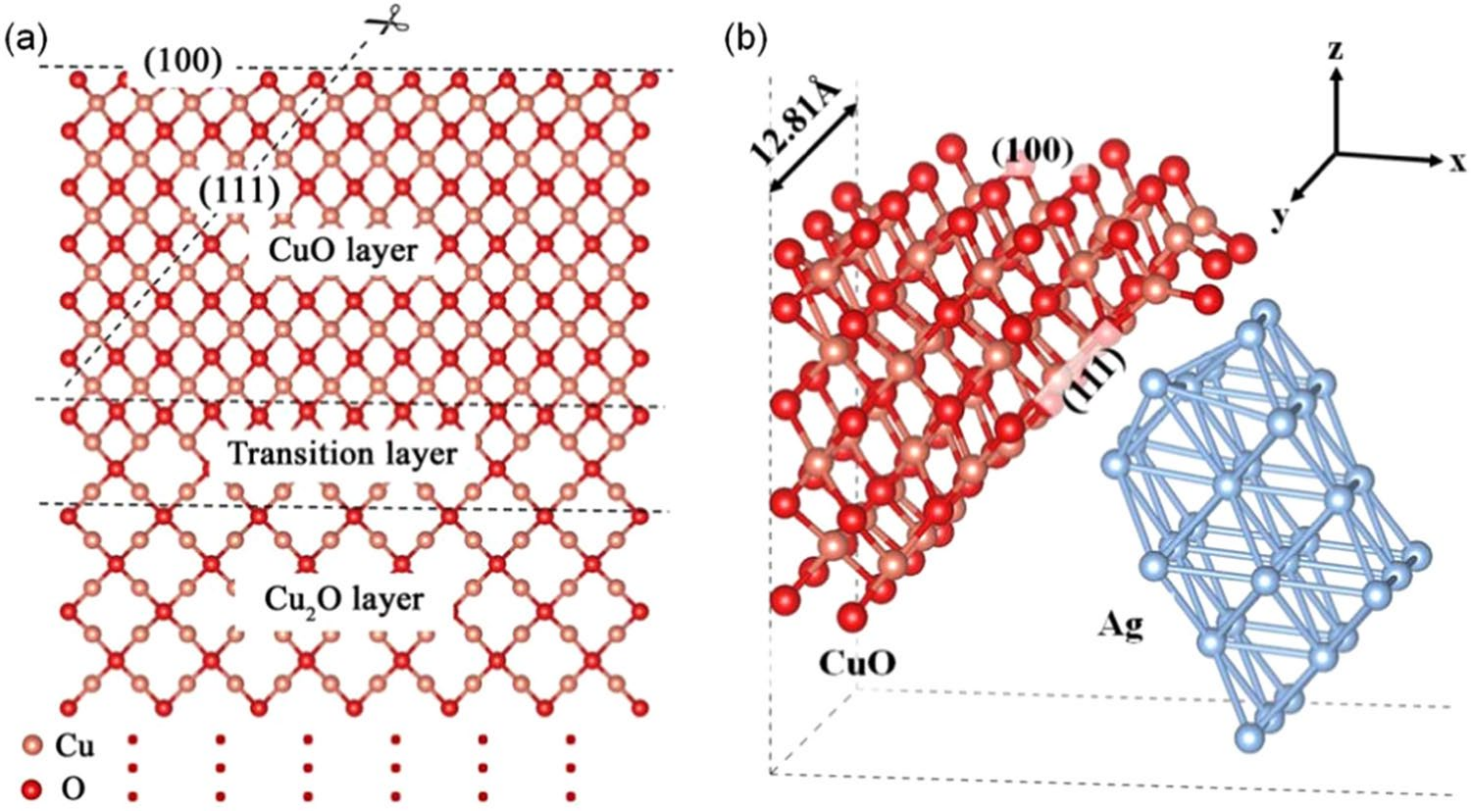
Construction of CuO* model and the CuO/Ag interface. (**a**) Structural model of the CuO/Cu_2_O(100) interface. (**b**) Structural model of the CuO(111)/Ag(111) interface.

The Cu_2_O/CuO(100) 6 × 3 supercell structure (the CuO surface was evolved from the Cu_2_O(100) surface) in Fig. 2a consists of 9 layers of Cu_2_O(100), 12 layers of CuO, and 4 transition layers in between^14^, with the structure being cleaved along the direction of the scissors as indicated. As Cu_2_O is a cubic lattice, we assume that the CuO lattice evolved from it shares the same cubic geometry, thus we set the cleavage angle to 35.26°($ta{n}^{-1}\sqrt{2}$) to ensure that the newly formed surface is a CuO(111) facet. A 4 layer Ag cluster derived from a pure Ag(111) surface model was used to simulate the silver substrate. Then we combined the cleaved Cu_2_O/CuO supercell and the Ag cluster together to mimic the CuO/Ag composite structure. As shown in Fig. 2b, such a unique interfacial configuration only has periodicity in the y-direction with a lattice constant of 12.81 Å, while in the x and z directions we add ~15 Å vacuum layers to avoid artificial interactions between slabs. It is noteworthy that the experimentally-determined thickness of the CuO shell is approximately 6 to 10 nm^14^, indicating that the CuO(111) and (100) surfaces are minimally influenced by Cu_2_O. Therefore our final simplified CuO/Ag model does not contain Cu_2_O. During relaxation all the atoms were relaxed except for the bottom 2 layers.

## Results and Discussion

### Formation of the interfacial charge polarization

**Figure 3:**
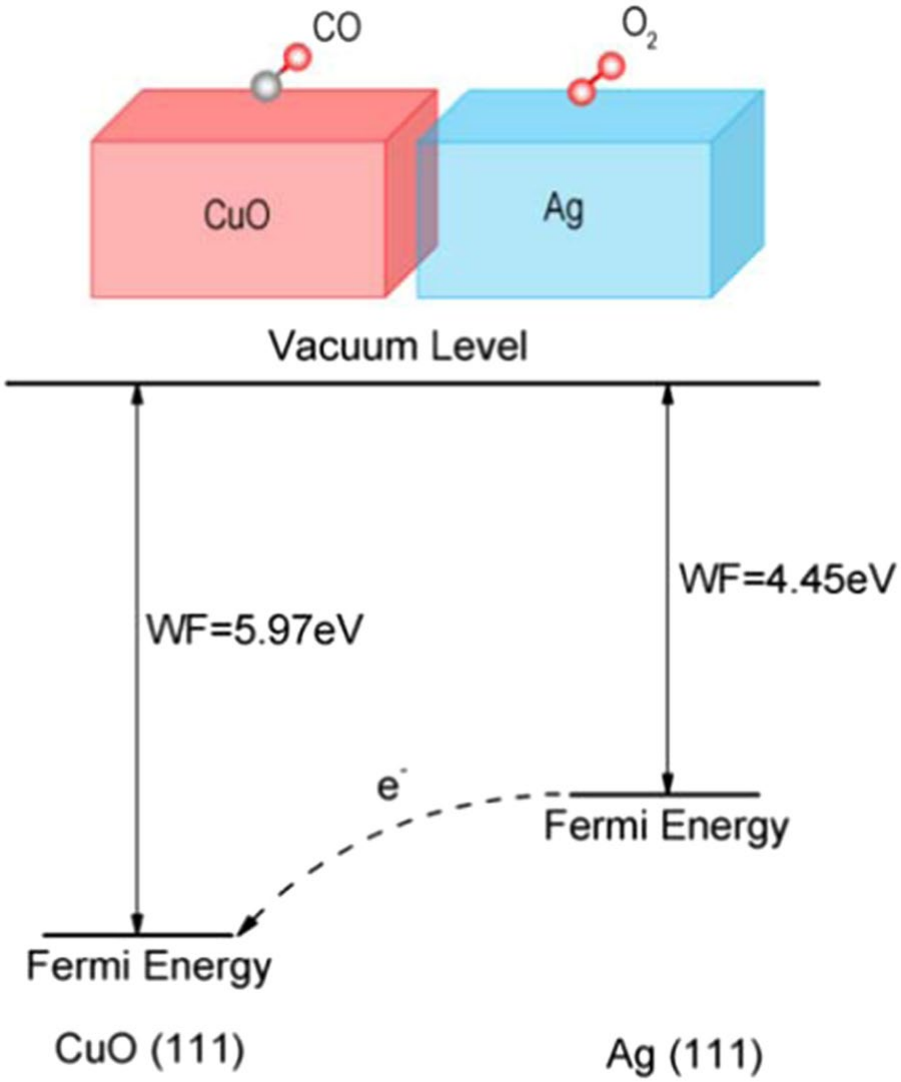
Diagram of the mechanism of charge transfer induced by the difference in work functions between the CuO(111) and Ag(111) surfaces.

According to the Schottky-Mott rule^24^, a Schottky barrier should be formed at the metal-semiconductor junction due to the difference in work function between the two materials^25^. In our case, because the work functions of Ag(111) and CuO(111) differ, surface polarization is inevitably induced at the CuO/Ag interface, as shown in Fig. 3. The work function of CuO(111) (5.97 eV) is much higher than that of the Ag(111) substrate (4.45 eV), indicating that the Fermi level of pure CuO is much lower than that of pure Ag. As a consequence, after the combination of these two materials, electrons should transfer from the one with a higher potential level to the material with a lower one, that is from Ag(111) to CuO(111) through the interface, thus generating electron-hole pairs. The effective separation of electrons and holes is usually referred to as *interfacial polarization*.

**Figure 4:**
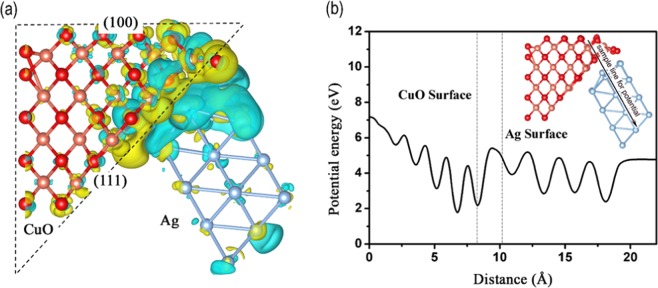
Charge transfer at the CuO/Ag interface. (**a**) Computed spatial distributions of charge density differences at the CuO(111)/Ag(111) interface. Here olive and cyan colors represent an increase (olive color) or a decrease (cyan color) in the electronic charge distribution. (**b**) Variations in static potential along the line traversing the Ag(111)/CuO interface, in the direction of the arrow.

Such a charge polarization is shown in Fig. 4a. Electrons should transfer from Ag (cyan colour) to CuO (olive colour) owing to the interfacial polarization effect driven by the work function difference at the interface. The number of transferred electrons is calculated by Bader charge analysis to be 2.29 e^−^. To further elucidate the electrostatic potential difference at the interface, the static potential surface along the direction perpendicular to the interface is given in Fig. 4b. Obviously the potential energy of Ag is higher than that of CuO along the sampling line, indicating the direction of the Schottky junction so formed. Through this junction, electrons can easily transfer from Ag to CuO but return again only with difficulty; by this means effective charge separation and charge polarization at the interface is achieved. It is worth mentioning that due to the limitation of computing power, we only use the small Ag cluster model derived from Ag(111) surface in this work. To ensure the validity of the charge transfer trend, we also performed a test calculation by using a 3-layers Ag(111) surface model as the substrate (Fig. S1). Results show a consistent trend that electrons still transfer from Ag to CuO even at large Ag(111) substrate. It should be noted that the conclusions made in this work are based on the interactions between O_2_ molecule and clean unoxidized Ag surface. The real O_2_/Ag interactions could be far more complicated due to the formation of the epitaxial Ag_2_O thin oxide overlayers on the surface of Ag^26^, which could affect the overall catalytic activities. To estimate its impact, we artificially added an Ag_3_O cluster to mimic the Ag_2_O layer in the middle between CuO and Ag as shown in Fig. S2. Results show that although the direction of charge transfer keeps unchanged, the transferred Bader charge decreases from 2.29 to 1.32 e^−^, indicating that the oxidation layer could reduce the catalytic activity to some extent. Therefore, we strongly suggest that the surface oxidation layer of Ag should be removed first when preparing the CuO/Ag catalyst experimentally.

### Charge transfer inside the CuO lattice

**Figure 5:**
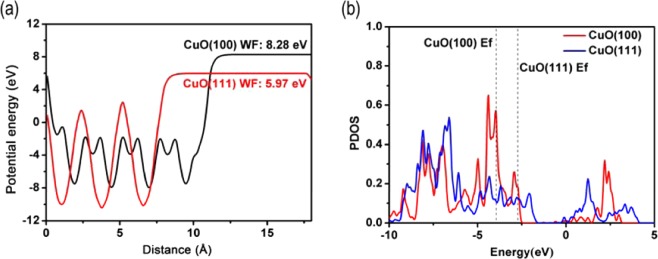
WF and PDOS of CuO(100) and (111) facets. (**a**) Computed potential energy of CuO(100) and (111) facets along z-axis direction. The Fermi level is alighed at zero. Work functions are the convergent values of these curves. (**b**) Computed partial density of states of isolated CuO(100) and (111) surface atoms.

After the interfacial polarization forms, a new question arises: how do electrons transfer inside the CuO lattice? As work function is defined as the thermodynamic work required to remove an electron from a material to the vacuum near its surface, a crystal facet with a higher work function should be more likely to accumulate electrons^25,27^. As shown in Fig. 5a, the work function of the CuO(100) facet (8.28 eV) is much higher than that of the (111) facet (5.97 eV), indicating that electrons are more likely to accumulate on CuO(100). To further illustrate this point, we performed partial density of states (PDOS) calculations of isolated CuO(100) and (111) surface atoms (Fig. 5b). The results show that CuO(100) and (111) have very similar electronic structures except for a very strange “shift” — similar electronic orbitals while different orbital occupations, leading to a difference in their Fermi levels. After electron charge injection resulting from external electron donation, electrons tend to transfer from a facet with a higher Fermi level to one with a lower one, that is from CuO(111) to (100). Eventually, the polarized charge due to interfacial polarization accumulates on the (100) surface, a property that affects the catalytic activity of the system. It is wort mentioning that the metallic properties the surfaces are caused by the unsaturated atoms on the surface, the CuO and Cu_2_O bulk structures are semiconductors according to previous reports^13,14^.

### Effect of charge polarization on catalytic activity

**Figure 6:**
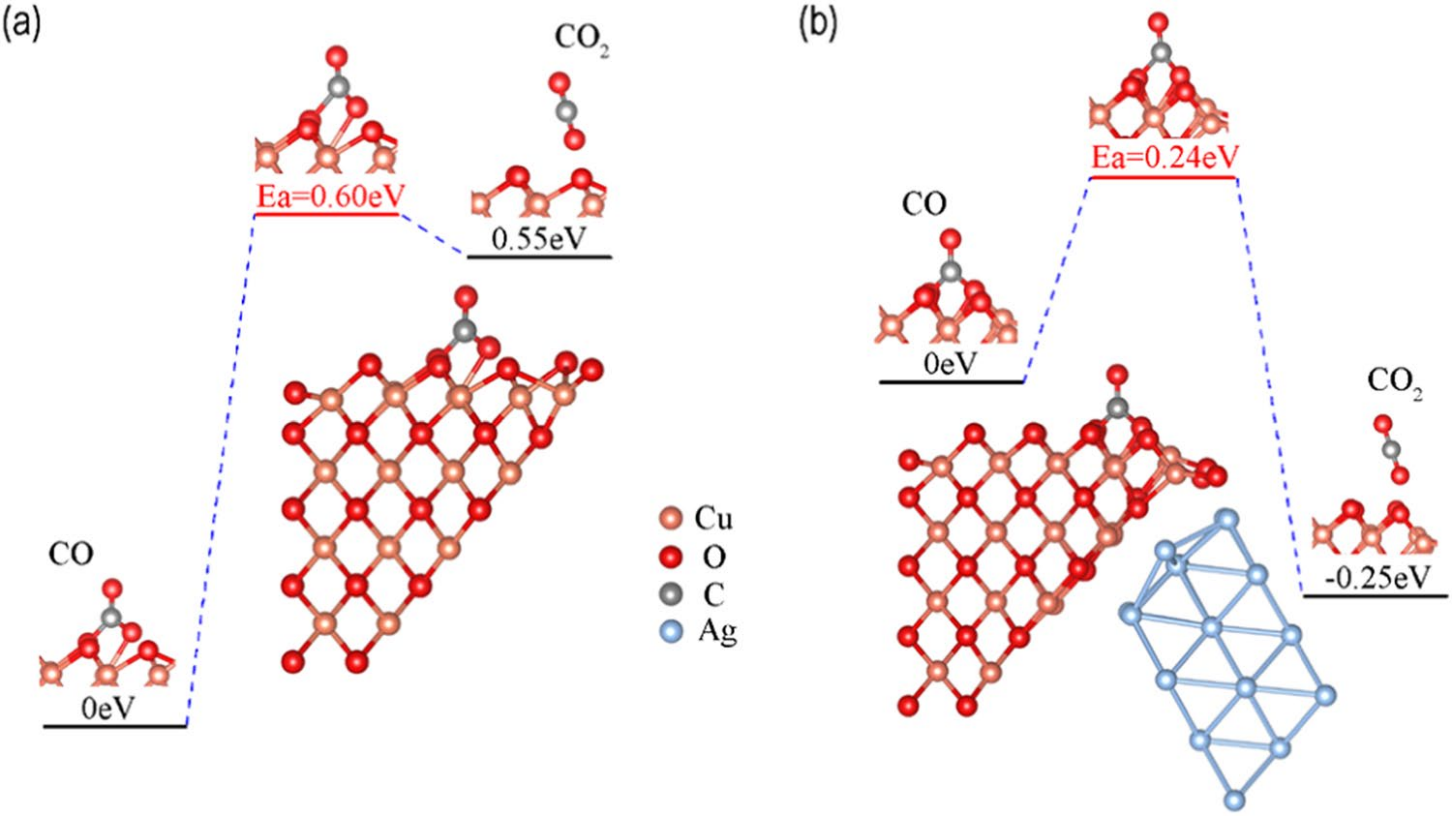
Minimum energy path for CO oxidation on CuO(100) surface in the (**a**) CuO* and (**b**) CuO/Ag systems.

Having computed the polarized charge accumulation on the CuO(100) surface, we now move on to study its impact on catalytic activity. CO oxidation on the CuO surface follows the Mars-Van Krevelen (M-vK) mechanism^28,29^. The CuO(100) surface is terminated by two-coordinated oxygen (O_2c_) atoms, which can easily combine with CO to form a CO_3_ surface intermediate. The CO_3_ intermediate easily decomposes to produce CO_2_, leaving an oxygen vacancy (*Vo*) at the active site. In order to evaluate the role of Ag in the CO oxidation, we built a new model CuO* by intentionally removing the Ag substrate from the CuO/Ag model as shown in Fig. 6a. Transition state search results (Fig. 6) reveal that the polarization charge accumulation induced by the Ag substrate dramatically lowers the barrier of CO oxidation from 0.60 eV to 0.24 eV. This indicates that interfacial polarization indeed enhances catalytic activity. Since it is suggested that the removal of the surface lattice oxygen is the rate-determining step of CO oxidation for the M-vK mechanism^13,28,29^, we compared our result (0.24 eV) with the barriers of rate-determining steps in other relevant systems, such as CO oxidation on Cu_2_O (111) (1.63 eV)^30^, CuO/Cu_2_O (110) (0.60 eV)^31^, CuO/Cu_2_O (100) (1.15 eV)^14^, and CuO/Cu_2_O (111) (0.37 eV)^14^, illustrating the superiority of our CuO/Ag model.

To further understand the enhancement of catalytic activity, we then calculated the CO adsorption energy on a CuO(100) surface in both the CuO* and CuO/Ag models. Normally, optimum activity is obtained at an intermediate adsorption energy according to the volcano shape rule^32,33^. Our results show that the CO adsorption energy in the CuO* system is 3.19 eV while in CuO/Ag it is 2.94 eV, indicating that the polarized charge drags the adsorption energy down closer to an optimum value, thereby enhancing catalytic activity.

In addition, the effect of polarization charge on the lattice oxygen reactivity of CuO(100) was also carefully studied. In the presence of the Ag substrate, the activation energy of a lattice oxygen decreases from 5.34 eV to 5.11 eV (Fig. S3), and the energy required to form an oxygen vacancy decreases from 5.68 eV to 5.36 eV. These changes in energy indicate that the injection of polarized charge can significantly assist activating lattice oxygen atoms, suggesting better catalytic activity.

### Effect of charge polarization on *V*_*O*_ recovery

After CO oxidation, the refilling of *V*_*O*_ is vital in order to enable the next reaction cycle. Our adsorption energy calculations (Table 2) indicate that in the presence of the Ag substrate, the O_2_ adsorption energy at the oxygen vacancy site on the CuO(111) surface increases from 1.45 eV to 1.64 eV, which should result in a significant improvement in catalytic recovery. CO cannot compete with O_2_ during the recovery process due to its much lower adsorption energy. Also, because O_2_ adsorption on Ag is weak, O_2_ prefers adsorption at the *V*_*O*_ site and thus readily heals the defect there. Such a feature agrees well with our previous experimental work^13^.

**Table 2:** Calculated O_2_ and CO adsorption energy Ead for CuO* and CuO/Ag with and without a surface oxygen vacancy, and for Ag with and without holes.

Species	CuO*(*Vo*)	CuO(*Vo*)/Ag	CuO*	CuO/Ag	Ag	Ag^+^
Ead^O2^ (eV)	1.45	1.64	0.00	0.01	0.01	0.28
Ead^CO^ (eV]	0.96	1.11	2.80	2.94	0.12	0.66

**Figure 7:**
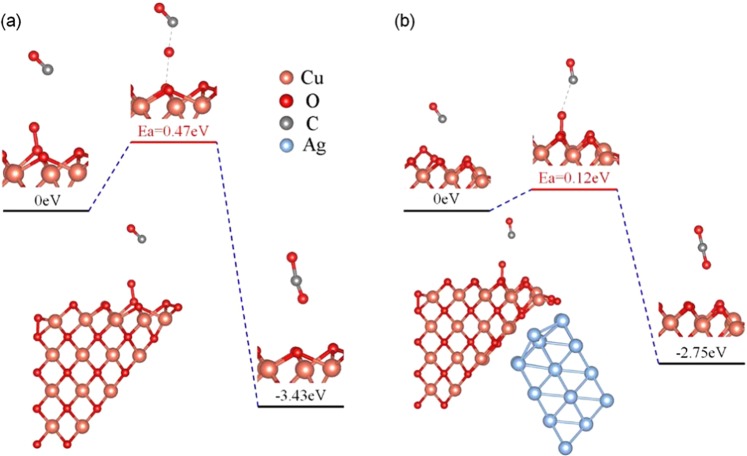
Minimum energy path for CO oxidation at the redundant oxygen site on CuO(100) surface in the (**a**) CuO* and (**b**) CuO/Ag systems.

After O_2_ has reoccupied the oxygen vacancy, there should be one additional oxygen atom bound to the CuO(100) surface. This redundant oxygen atom can be much more active than a lattice oxygen^14^. We calculated the desorption activation energy by removing a redundant oxygen atom gradually from the CuO(100) surface in both CuO* and CuO/Ag models as shown in Fig. S4. The resulted desorption energies are much lower than the lattice oxygen activation energies (Fig. S3), suggesting that the activation of the redundant oxygen should be much easier than the lattice oxygen. Moreover, NEB simulations further demonstrated that the reaction between CO and the redundant oxygen is not the rate-determine step in the reaction cycles on both CuO* and CuO/Ag due to the very low barriers of 0.47 and 0.12 eV respectively as shown in Fig. 7.

### Effect of charge polarization on the Ag substrate

**Figure 8:**
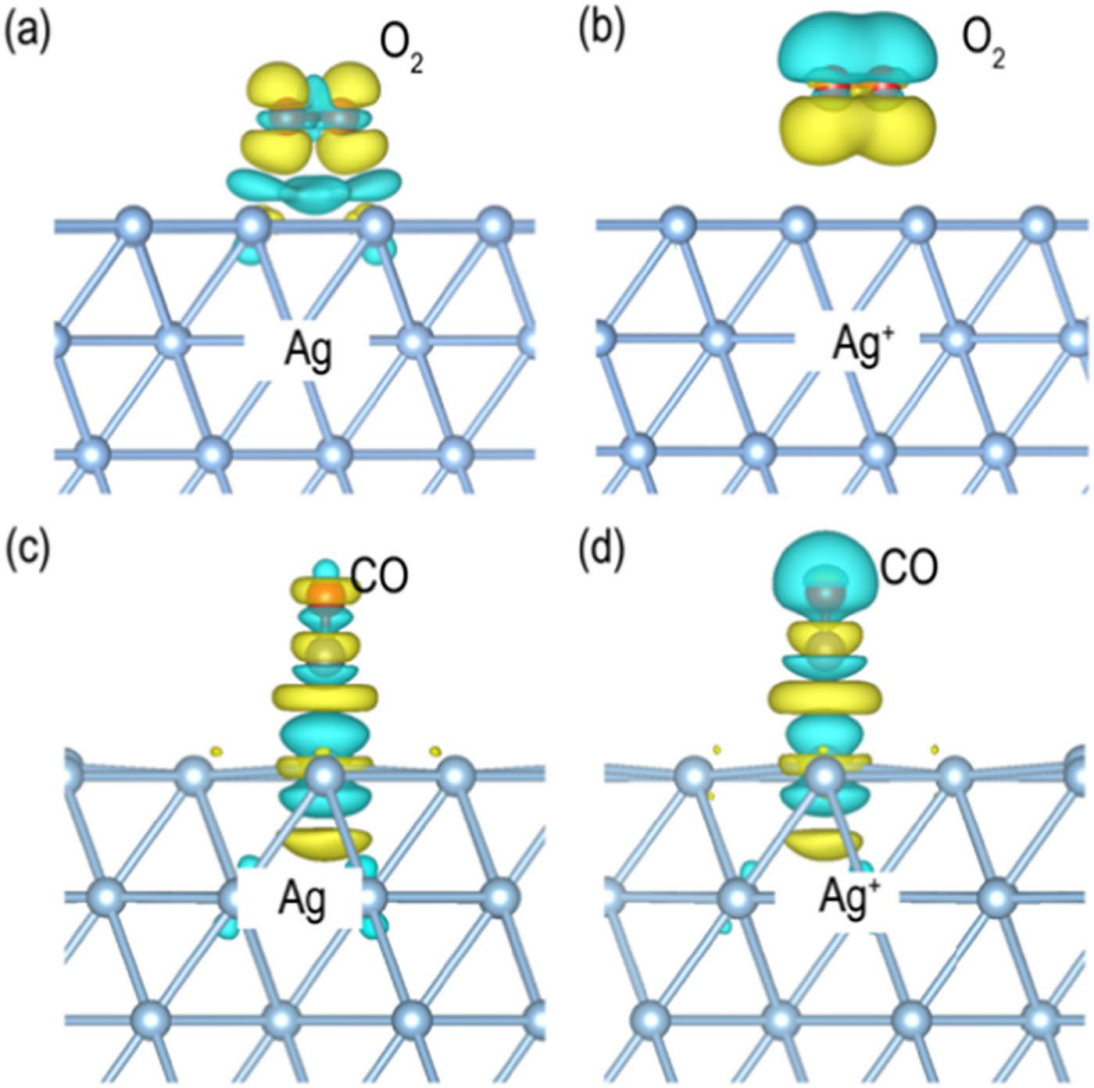
Differential charge densities for O_2_/CO adsorption on Ag/Ag+. (**a**) O_2_ adsorption on Ag; (**b**) O_2_ adsorption on Ag^+^; (**c**) CO adsorption on Ag; (**d**) CO adsorption on Ag^+^. After adsorption, electrons transfer from the cyan areas to the olive areas.

In addition to CuO, we also focused on the impact of charge polarization on the Ag substrate. After combining CuO and Ag, holes should remain on Ag(111) as mentioned above. Compared with neutral Ag, Ag with a hole (Ag^+^) has stronger adsorption energies for both O_2_ and CO (Table 2), which may result from the charge redistributions. The neutral Ag donates 0.41 e^−^ electrons to O_2_ after adsorption while Ag^+^ has almost no charge exchange with O_2_. However, Ag^+^ can induce a significant charge redistribution on O_2_ compared with Ag as depicted in Fig. 8a,b. The electrons that originally away from Ag substrate would accumulate in the middle between Ag and O_2_ after O_2_ adsorption. These redistributed electrons thus form a strong electrostatic interaction with the positive charge on Ag^+^, increasing the adsorption energy from 0.01 to 0.28 eV (Table 2). A similar phenomenon occurs in the case of CO adsorption (Fig. 8), wherein neutral Ag donates 0.09 e^−^ electrons to CO during adsorption while Ag^+^ receives 0.05 e^−^ from CO; the charge redistribution in CO enhances the electrostatic attraction between Ag and CO, and thus increases the adsorption energy from 0.12 to 0.66 eV.

**Figure 9:**
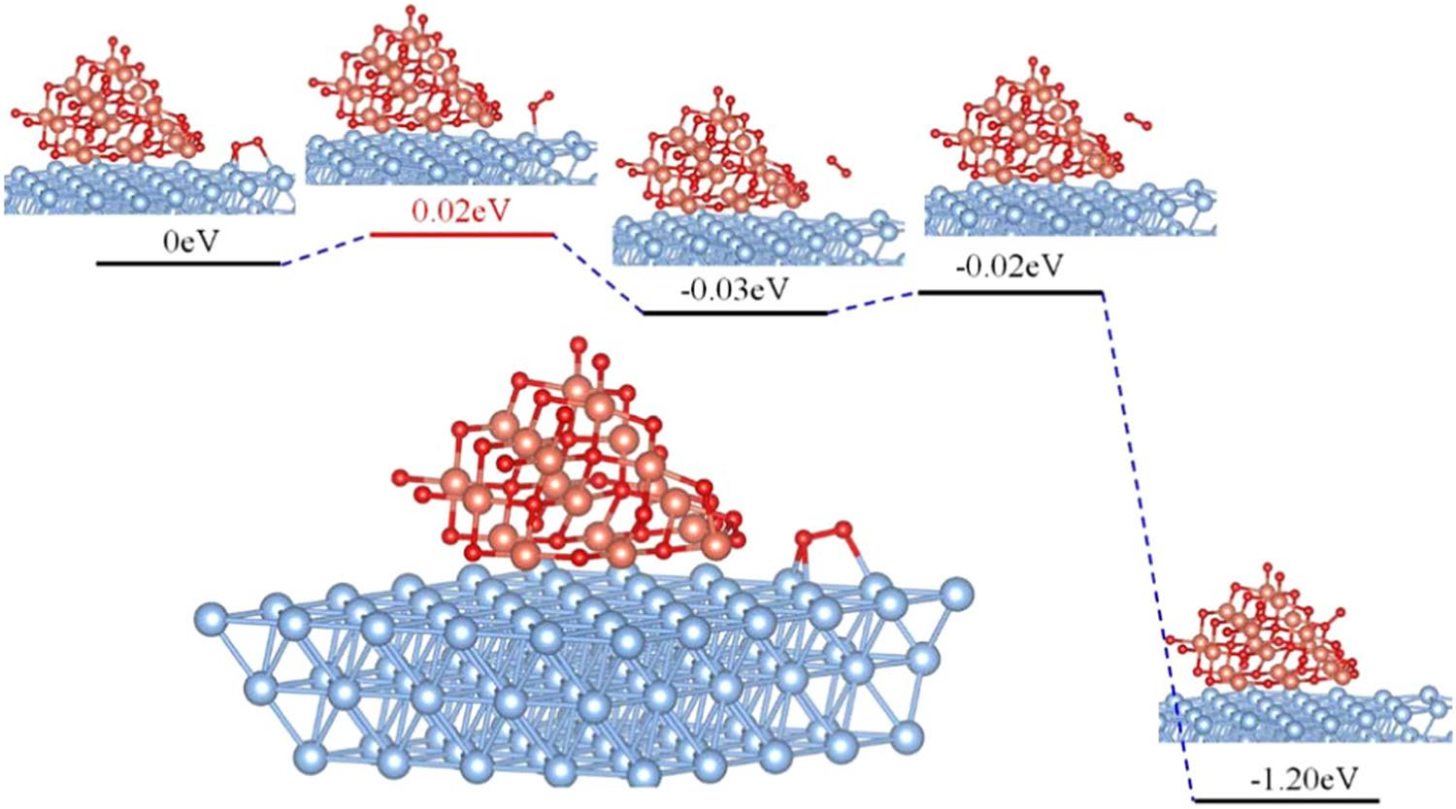
Minimum energy path for O_2_ migration from Ag(111) surface to the *V*_*O*_ site on CuO(100).

**Figure 10:**
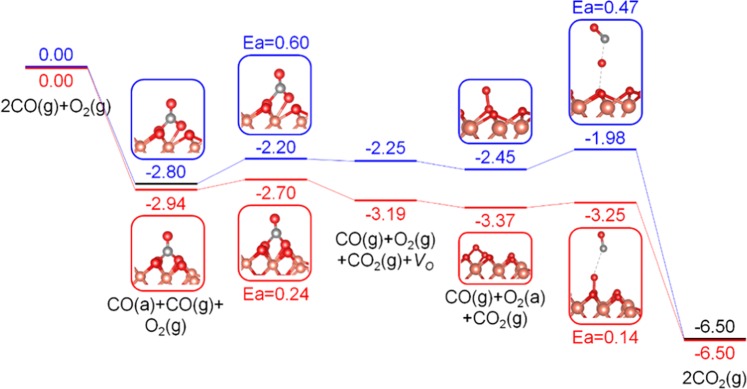
Energy profile of each elementary step in CO oxidation catalyzed by CuO* (blue lines) and CuO/Ag (red lines).

Keeping these adsorption energies in mind (Table 2), we propose a reasonable conjecture, a synergistic effect that encompasses the whole catalyst recovery process. Before CO oxidation, CO tends to adsorb on the CuO surface, while O_2_ prefers to adsorb on the Ag substrate; this differential preference is due to their different adsorption energies. After the creation of *V*_*O*_ site on CuO surface, at certain temperatures and pressures, in addition to obtaining oxygen from the air, it is also possible for *V*_*O*_ sites to obtain oxygen from Ag substrate due to the short distance between CuO and Ag, and the huge difference in O_2_ adsorption energies of these two materials. The barrier of O_2_ migration from Ag surface to CuO *V*_*O*_ site was studied by using the larger model in Fig. S1. Due to the limitation of the computing power, here a lower level of accuracy (cutoff energy = 300 eV) was applied and only the atoms that are directly bind with O_2_ are allowed to relax during the NEB calculations. The potential energy surface of O_2_ migration (Fig. 9) shows a negligible barrier of 0.02 eV, indicating that O_2_ migration is readily to occur at ambient conditions. Overall, the Ag substrate acts as an O_2_ store, which collects O_2_ molecules before catalytic reaction, and then delivers these to CuO once *V*_*O*_ forms, thus facilitating CuO recovery. The energy profile of each elementary step in CO oxidation catalyzed by CuO* (blue lines) and CuO/Ag (red lines) are summarized as shown in Fig. 10, showing that the presence of Ag substrate can not only significantly lower down the barriers of CO oxidation, but also promote the recovery of the surface *V*_*O*_ site, making the catalyst more efficient.

## Conclusion

In summary, by utilizing an interfacial polarization effect, we have designed a CuO/Ag heterogeneous catalyst for CO oxidation with very promising catalytic activity. The significant improvement in catalytic performance is ascribed to the accumulation of polarization charge on the CuO surface that is induced by the work function difference between CuO and Ag. Interestingly, charge polarization can not only promote CO oxidation, but also promote the healing of the *V*_*O*_ defect, making the catalyst more durable. These new insights from the case of CuO/Ag can be helpful for studying how the polarization effect may improve other catalytic systems, providing a new approach toward the rational design of more efficient heterogeneous catalysts.

## Supplementary information

Supporting Information.
