# Supplementary material for: Carbon Monoxide Oxidation Promoted by Surface Polarization Charges in a CuO/Ag Hybrid Catalyst
Source: Sci Rep. 2020 Feb 13;10:2552. doi: 10.1038/s41598-020-59531-0 (PMC7018725; doi:10.1038/s41598-020-59531-0)
Supplement: Supplementary file 1 — Supporting Information. [file 41598_2020_59531_MOESM1_ESM.docx]

**Supporting Information**

**Carbon Monoxide Oxidation Promoted by Surface Polarization Charges in a CuO/Ag Hybrid Catalyst**

Xijun Wang^1,2^†, Chuanyi Jia^3^†, Edward Sharman^4^, Guozhen Zhang^1^, Xin Li^1^*, Jun Jiang^1^

^1^Hefei National Laboratory for Physical Sciences at the Microscale, CAS Key Laboratory of Mechanical Behavior and Design of Materials, School of Chemistry and Materials Science, University of Science and Technology of China, Hefei, Anhui 230026, P. R. China

^2^Department of Chemical and Biomolecular Engineering, North Carolina State University, Raleigh, North Carolina 27606, USA

^3^Guizhou Provincial Key Laboratory of Computational Nano-Material Science, Institute of Applied Physics, Guiyang 550018, China

^4^Department of Neurology, University of California, Irvine, California 92697, USA

*Corresponding Authors: [lix212@ustc.edu.cn](mailto:lix212@ustc.edu.cn)

†These authors contributed equally to this work.


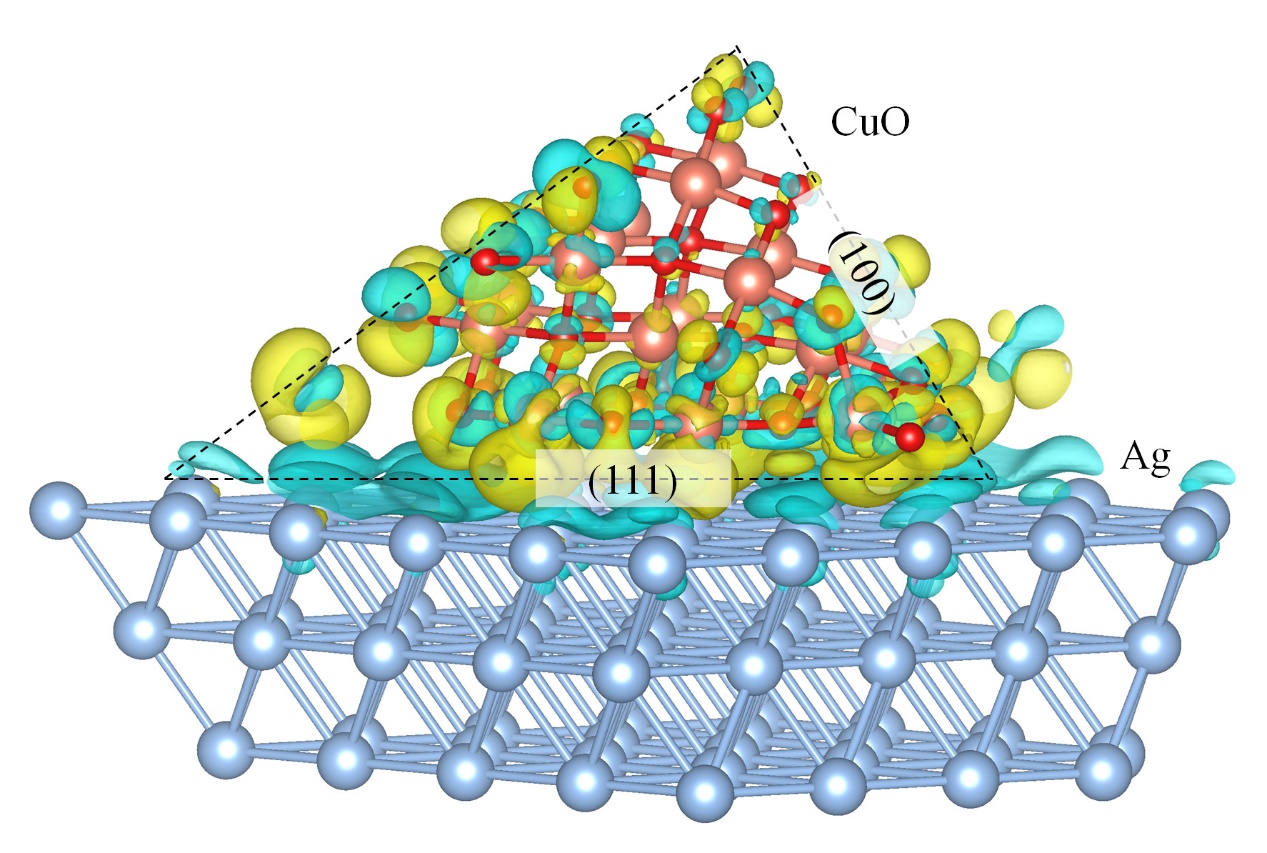


**Figure S1 | Computed spatial distributions of charge density differences at the CuO(111)/Ag(111) interface.** Here a 3-layers Ag (111) surface model was used to combine with CuO*. Olive and cyan colors represent an increase (olive color) or a decrease (cyan color) in the electronic charge distribution.


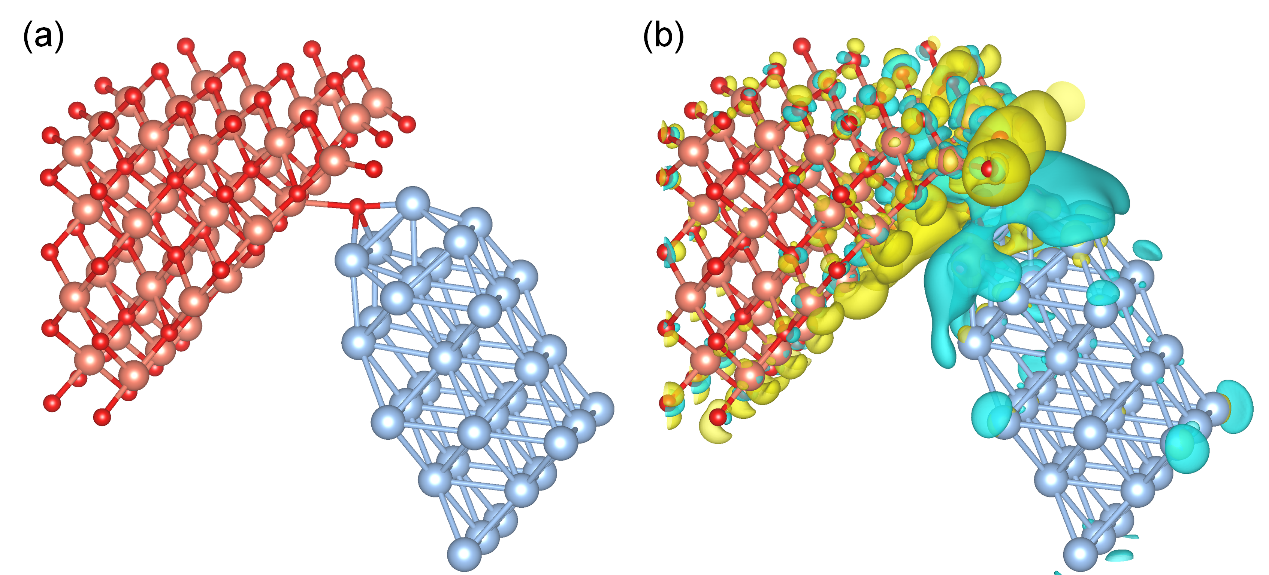


**Figure S2 | Computed spatial distributions of charge density differences at the CuO(111)/Ag_3_O/Ag(111) interface.** Here an Ag_3_O cluster is added in the middle between CuO and Ag to mimic the Ag_2_O thin oxide overlayer on the surface of Ag.^S1^


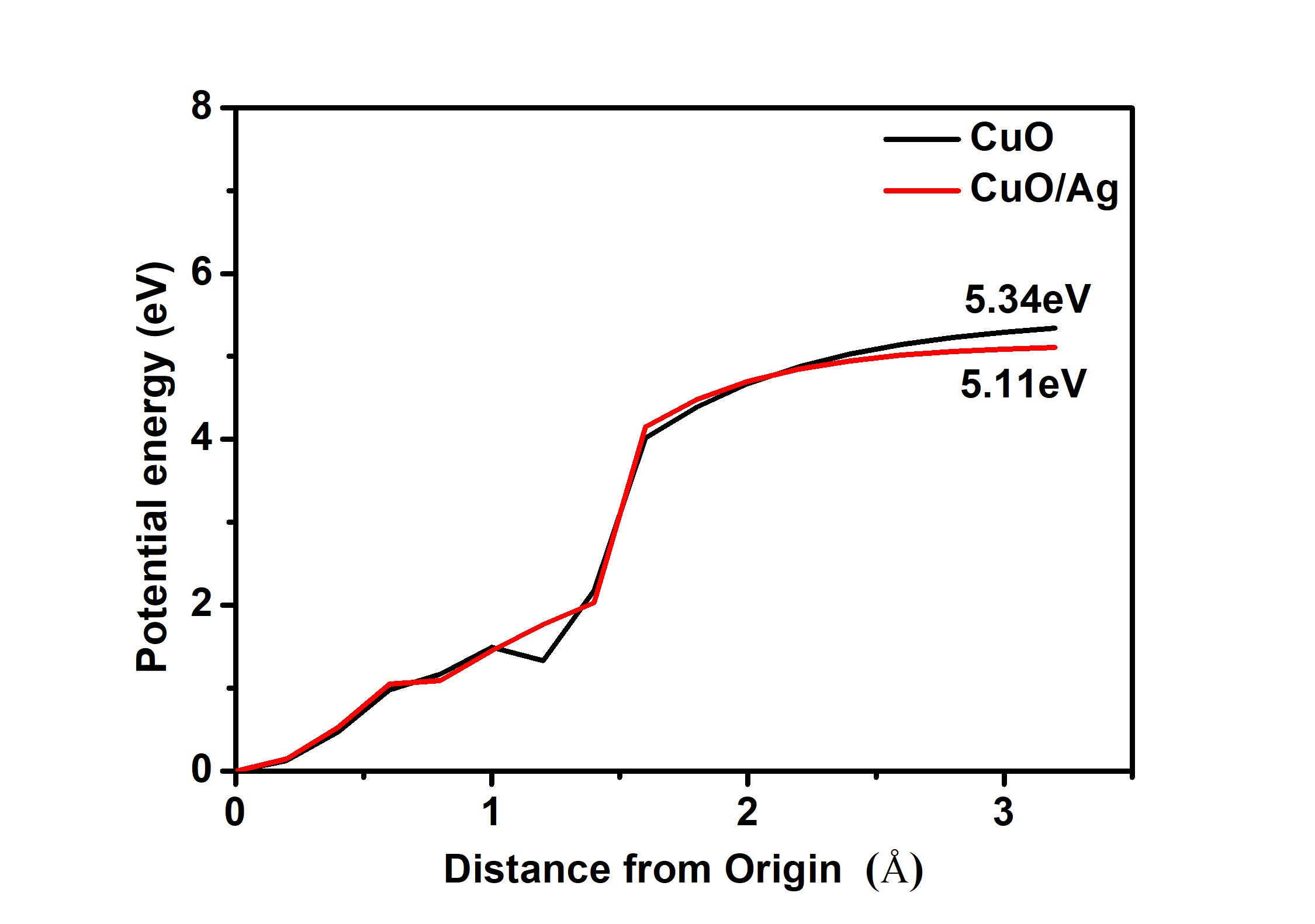


**Figure S3 | Lattice oxygen activation energy for the CuO* and CuO/Ag systems, respectively.**


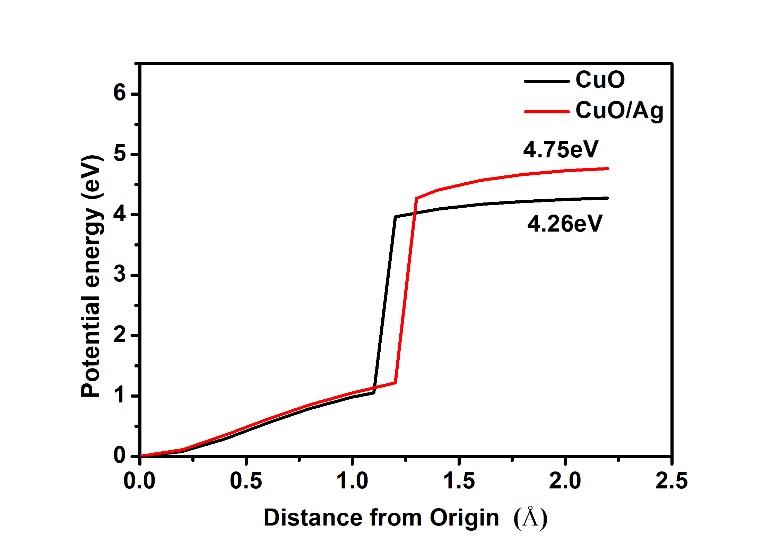


**Figure S4 | Desorption activation energy of the redundant oxygen atom on CuO* and CuO/Ag surface respectively.**

**Cartesian coordinates (VASP output CONTCARs):**

1. **CuO/Ag interface.**

1.0

40.0000000000 0.0000000000 0.0000000000

0.0000000000 12.8087997437 0.0000000000

0.0000000000 0.0000000000 30.0000000000

Cu O Ag

42 60 30

Direct

0.026629999 0.085330001 0.266339986

0.030920002 0.088129998 0.430559985

0.027430001 0.253259996 0.351010005

0.026910001 0.250710018 0.181549994

0.080669999 0.086230000 0.351049995

0.127880001 0.077169999 0.265100002

0.136580002 0.096870002 0.434829998

0.080390000 0.250860007 0.266410001

0.083849996 0.262279987 0.435409991

0.134169996 0.253259996 0.351010005

0.075520003 0.082570006 0.179600000

0.181900001 0.097730000 0.345429993

0.244689989 0.138699994 0.421960004

0.189759994 0.270139993 0.430029996

0.026629999 0.418660003 0.266339986

0.031009999 0.421400007 0.430480003

0.027430001 0.586589978 0.351010005

0.026910001 0.584049996 0.181549994

0.080669999 0.419559973 0.351049995

0.132660007 0.422569987 0.262719981

0.137520003 0.430209999 0.434520022

0.080390000 0.584190008 0.266410001

0.084259999 0.596010014 0.435529995

0.134169996 0.586589978 0.351010005

0.075819999 0.415640012 0.179759995

0.186609995 0.435149989 0.349599997

0.239860010 0.479290001 0.440939999

0.187110007 0.610870002 0.435730012

0.026629999 0.751990023 0.266339986

0.031029999 0.754469958 0.430469990

0.027430001 0.919930013 0.351010005

0.026910001 0.917379942 0.181549994

0.080669999 0.752889956 0.351049995

0.130299997 0.746949966 0.263590002

0.136010003 0.765770010 0.434749985

0.080390000 0.917519991 0.266410001

0.083679998 0.930190008 0.435409991

0.134169996 0.919930013 0.351010005

0.075790000 0.748640012 0.179629993

0.186479998 0.757690045 0.346439997

0.237809992 0.783789964 0.432639980

0.187709999 0.933690045 0.432489999

0.000830000 0.169970013 0.393159993

0.000940000 0.200870002 0.438090007

0.000530000 0.169450004 0.309099992

0.999930000 0.167600011 0.223680003

0.000230000 0.167140012 0.139129988

0.053920001 0.002820000 0.308629990

0.060460001 0.027699999 0.470609983

0.107570004 0.169970013 0.393159993

0.114830005 0.193500000 0.471050008

0.054189998 0.003200000 0.393180021

0.107270002 0.169450004 0.309099992

0.053679997 0.000090000 0.139080000

0.101460004 0.152129988 0.222720003

0.053429997 0.000810000 0.224249999

0.155799997 0.011649999 0.306849988

0.167089999 0.028010000 0.470490011

0.209940004 0.184609988 0.380029996

0.219390011 0.201040001 0.463460000

0.160929990 0.003200000 0.393180021

0.282019997 0.077040001 0.418740018

0.000830000 0.503310029 0.393159993

0.001050000 0.534099993 0.438100020

0.000530000 0.502780024 0.309099992

0.999930000 0.500930013 0.223680003

0.000230000 0.500469995 0.139129988

0.053920001 0.336149989 0.308629990

0.060610002 0.360790001 0.470450020

0.107570004 0.503310029 0.393159993

0.116899991 0.530439990 0.470140012

0.054189998 0.336530005 0.393180021

0.107270002 0.502780024 0.309099992

0.053679997 0.333429975 0.139080000

0.101880002 0.487919999 0.222230005

0.053429997 0.334140012 0.224249999

0.162129998 0.356720013 0.303859997

0.169729996 0.362800015 0.469410006

0.209590006 0.518939990 0.391950003

0.217199993 0.566869965 0.475249990

0.160929990 0.336530005 0.393180021

0.270079994 0.387939990 0.425909996

0.000830000 0.836640012 0.393159993

0.001100000 0.867359988 0.438140011

0.000530000 0.836109969 0.309099992

0.999930000 0.834259996 0.223680003

0.000230000 0.833809992 0.139129988

0.053920001 0.669489986 0.308629990

0.060839999 0.694440027 0.470440006

0.107570004 0.836640012 0.393159993

0.114730000 0.863160003 0.471150017

0.054189998 0.669870002 0.393180021

0.107270002 0.836109969 0.309099992

0.053679997 0.666760033 0.139080000

0.101539993 0.818860025 0.222829994

0.053429997 0.667480009 0.224249999

0.160160005 0.676930013 0.303809992

0.166209996 0.705450004 0.472450002

0.209780002 0.842900007 0.386440023

0.215210009 0.865510014 0.469700019

0.160929990 0.669870002 0.393180021

0.268830013 0.696139975 0.416720009

0.267109990 0.382569987 0.097429999

0.267109990 0.611519991 0.097429999

0.298300004 0.497049996 0.023890001

0.235929990 0.268099993 0.170959997

0.298300004 0.268099993 0.023890001

0.235929990 0.497049996 0.170959997

0.277579999 0.382569987 0.234850009

0.339949989 0.382569987 0.087779999

0.350419998 0.382569987 0.225209999

0.204279995 0.387199985 0.249699990

0.381610012 0.268099993 0.151669995

0.308769989 0.497049996 0.161320003

0.234980011 0.287339978 0.328049978

0.308769989 0.268099993 0.161320003

0.319239998 0.268099993 0.298740005

0.235929990 0.725990023 0.170959997

0.298300004 0.726000000 0.023890001

0.339949989 0.611519991 0.087779999

0.350419998 0.611519991 0.225209999

0.201909995 0.622850011 0.252680000

0.277579999 0.611519991 0.234850009

0.381610012 0.726000000 0.151669995

0.319239998 0.497049996 0.298740005

0.247230005 0.726989949 0.310429986

0.381610012 0.497049996 0.151669995

0.308769989 0.726000000 0.161320003

0.319239998 0.725990023 0.298740005

0.245169997 0.500599993 0.309910011

0.292430019 0.381019991 0.366070016

0.289330006 0.609749981 0.367009989

1. **Transition state structure in Figure 6a.**

1.0

40.0000000000 0.0000000000 0.0000000000

0.0000000000 12.8087997437 0.0000000000

0.0000000000 0.0000000000 30.0000000000

Cu O C

42 61 1

Direct

0.026629999 0.085330001 0.132049990

0.032269999 0.091270000 0.295799987

0.027430001 0.253259996 0.216719993

0.026910001 0.250710018 0.047260002

0.080669999 0.086230000 0.216760000

0.130669999 0.084850002 0.131259990

0.140540004 0.110279996 0.299549993

0.080390000 0.250860007 0.132119997

0.086369997 0.270310010 0.300229994

0.134169996 0.253259996 0.216719993

0.080150002 0.083619993 0.047200000

0.181500006 0.101839997 0.214849997

0.226852989 0.106890003 0.302029991

0.190320003 0.277660003 0.298390007

0.026629999 0.418660003 0.132049990

0.032269999 0.424599993 0.295799987

0.027430001 0.586589978 0.216719993

0.026910001 0.584049996 0.047260002

0.080669999 0.419559973 0.216760000

0.130669999 0.418179994 0.131259990

0.140540004 0.443619984 0.299549993

0.080390000 0.584190008 0.132119997

0.086369997 0.603640012 0.300229994

0.134169996 0.586589978 0.216719993

0.080150002 0.416950004 0.047200000

0.181500006 0.435169980 0.214849997

0.226852989 0.440220013 0.302029991

0.190106010 0.610615032 0.299604003

0.026629999 0.751990023 0.132049990

0.032269999 0.757930013 0.295799987

0.027430001 0.919930013 0.216719993

0.026910001 0.917379942 0.047260002

0.080669999 0.752889956 0.216760000

0.130669999 0.751510014 0.131259990

0.138702011 0.767348001 0.301209005

0.080390000 0.917519991 0.132119997

0.085938996 0.934044003 0.304671987

0.134169996 0.919930013 0.216719993

0.080150002 0.750290001 0.047200000

0.181500006 0.768499963 0.214849997

0.226852989 0.773549996 0.302029991

0.190320003 0.944320006 0.298390007

0.000830000 0.169970013 0.258869998

0.002190000 0.203299996 0.303409990

0.000530000 0.169450004 0.174810012

0.999930000 0.167600011 0.089390000

0.000230000 0.167140012 0.004840000

0.053920001 0.002820000 0.174339994

0.064570004 0.040199999 0.334549999

0.107570004 0.169980009 0.258869998

0.122529995 0.218370002 0.332979997

0.054189998 0.003200000 0.258890009

0.107270002 0.169450004 0.174810012

0.053679997 0.000090000 0.004790000

0.106669998 0.167600011 0.089390000

0.053429997 0.000810000 0.089960003

0.156169999 0.016180000 0.174310001

0.179809999 0.065959999 0.330270004

0.207410002 0.179982001 0.254850006

0.228630018 0.251399989 0.337130006

0.160929990 0.003200000 0.258890009

0.249919987 0.332530005 0.328999996

0.000830000 0.503310029 0.258869998

0.002190000 0.536629961 0.303409990

0.000530000 0.502780024 0.174810012

0.999930000 0.500930013 0.089390000

0.000230000 0.500469995 0.004840000

0.053920001 0.336149989 0.174339994

0.064570004 0.373530005 0.334549999

0.107570004 0.503310029 0.258869998

0.122529995 0.551709999 0.332979997

0.054189998 0.336530005 0.258890009

0.107270002 0.502780024 0.174810012

0.053679997 0.333429975 0.004790000

0.106669998 0.500930013 0.089390000

0.053429997 0.334140012 0.089960003

0.156169999 0.349510014 0.174310001

0.179809999 0.399290001 0.330270004

0.207410002 0.513320043 0.254850006

0.228630018 0.584730028 0.337130006

0.160929990 0.336530005 0.258890009

0.249919987 0.665870002 0.328999996

0.000830000 0.836640012 0.258869998

0.002190000 0.869960018 0.303409990

0.000530000 0.836109969 0.174810012

0.999930000 0.834259996 0.089390000

0.000230000 0.833809992 0.004840000

0.053920001 0.669489986 0.174339994

0.064570004 0.706860025 0.334549999

0.107570004 0.836640012 0.258869998

0.124284005 0.865276003 0.342908001

0.054189998 0.669870002 0.258890009

0.107270002 0.836109969 0.174810012

0.053679997 0.666760033 0.004790000

0.106669998 0.834259996 0.089390000

0.053429997 0.667480009 0.089960003

0.156169999 0.682849974 0.174310001

0.166036987 0.755134986 0.359033998

0.207410002 0.846649989 0.254850006

0.228630018 0.918069987 0.337130006

0.160929990 0.669870002 0.258890009

0.249919987 0.999199985 0.328999996

0.147979009 0.857466011 0.419180997

0.149871004 0.820709999 0.383116023

1. **Transition state structure in Figure 6b.**

1.0

40.0000000000 0.0000000000 0.0000000000

0.0000000000 12.8087997437 0.0000000000

0.0000000000 0.0000000000 30.0000000000

Cu O Ag C

42 61 30 1

Direct

0.026629999 0.085330001 0.132049990

0.030920002 0.088129998 0.296270021

0.027430001 0.253259996 0.216719993

0.026910001 0.250710018 0.047260002

0.080669999 0.086230000 0.216760000

0.127880001 0.077169999 0.130809991

0.136580002 0.096870002 0.300540002

0.080390000 0.250860007 0.132119997

0.083849996 0.262279987 0.301120027

0.134169996 0.253259996 0.216719993

0.075520003 0.082570006 0.045299999

0.181900001 0.097730000 0.211129999

0.244689989 0.138699994 0.287669977

0.189759994 0.270139993 0.295740000

0.026629999 0.418660003 0.132049990

0.031009999 0.421400007 0.296190008

0.027430001 0.586589978 0.216719993

0.026910001 0.584049996 0.047260002

0.080669999 0.419559973 0.216760000

0.132660007 0.422569987 0.128419995

0.137520003 0.430209999 0.300220013

0.080390000 0.584190008 0.132119997

0.084259999 0.596010014 0.301239999

0.134169996 0.586589978 0.216719993

0.075819999 0.415640012 0.045469999

0.186609995 0.435149989 0.215299988

0.239080000 0.465009977 0.308189996

0.188830996 0.605087000 0.297795010

0.026629999 0.751990023 0.132049990

0.031029999 0.754469958 0.296180026

0.027430001 0.919930013 0.216719993

0.026910001 0.917379942 0.047260002

0.080669999 0.752889956 0.216760000

0.130299997 0.746949966 0.129299998

0.136010003 0.765770010 0.300460021

0.080390000 0.917519991 0.132119997

0.083679998 0.930190008 0.301110013

0.134169996 0.919930013 0.216719993

0.075790000 0.748640012 0.045330000

0.186479998 0.757690045 0.212149986

0.237809992 0.783789964 0.298350016

0.187709999 0.933690045 0.298189990

0.000830000 0.169970013 0.258869998

0.000940000 0.200870002 0.303789997

0.000530000 0.169450004 0.174810012

0.999930000 0.167600011 0.089390000

0.000230000 0.167140012 0.004840000

0.053920001 0.002820000 0.174339994

0.060460001 0.027699999 0.336320019

0.107570004 0.169970013 0.258869998

0.114830005 0.193500000 0.336749999

0.054189998 0.003200000 0.258890009

0.107270002 0.169450004 0.174810012

0.053679997 0.000090000 0.004790000

0.101460004 0.152129988 0.088429999

0.053429997 0.000810000 0.089960003

0.155799997 0.011649999 0.172549995

0.167089999 0.028010000 0.336200015

0.209940004 0.184609988 0.245740000

0.219390011 0.201040001 0.329169973

0.160929990 0.003200000 0.258890009

0.282019997 0.077040001 0.284449991

0.000830000 0.503310029 0.258869998

0.001050000 0.534099993 0.303809992

0.000530000 0.502780024 0.174810012

0.999930000 0.500930013 0.089390000

0.000230000 0.500469995 0.004840000

0.053920001 0.336149989 0.174339994

0.060610002 0.360790001 0.336159992

0.107570004 0.503310029 0.258869998

0.116899991 0.530439990 0.335839971

0.054189998 0.336530005 0.258890009

0.107270002 0.502780024 0.174810012

0.053679997 0.333429975 0.004790000

0.101880002 0.487919999 0.087940001

0.053429997 0.334140012 0.089960003

0.162129998 0.356720013 0.169570001

0.170519996 0.365469995 0.334881020

0.209590006 0.518939990 0.257649994

0.214239001 0.565038009 0.349869982

0.160929990 0.336530005 0.258890009

0.273162007 0.385784007 0.293397013

0.000830000 0.836640012 0.258869998

0.001100000 0.867359988 0.303849983

0.000530000 0.836109969 0.174810012

0.999930000 0.834259996 0.089390000

0.000230000 0.833809992 0.004840000

0.053920001 0.669489986 0.174339994

0.060839999 0.694440027 0.336149979

0.107570004 0.836640012 0.258869998

0.114730000 0.863160003 0.336859989

0.054189998 0.669870002 0.258890009

0.107270002 0.836109969 0.174810012

0.053679997 0.666760033 0.004790000

0.101539993 0.818860025 0.088540006

0.053429997 0.667480009 0.089960003

0.160160005 0.676930013 0.169520013

0.173403990 0.677454024 0.346208000

0.209780002 0.842900007 0.252149995

0.215210009 0.865510014 0.335399977

0.160929990 0.669870002 0.258890009

0.268830013 0.696139975 0.282429981

0.193110001 0.627559005 0.418769010

0.267109990 0.382569987 0.963139979

0.267109990 0.611519991 0.963139979

0.298300004 0.497049996 0.889599991

0.235929990 0.268099993 0.036670001

0.298300004 0.268099993 0.889599991

0.235929990 0.497049996 0.036670001

0.277579999 0.382569987 0.100560006

0.339949989 0.382569987 0.953490003

0.350419998 0.382569987 0.090920003

0.204279995 0.387199985 0.115400004

0.381610012 0.268099993 0.017379999

0.308769989 0.497049996 0.027030001

0.234980011 0.287339978 0.193759998

0.308769989 0.268099993 0.027030001

0.319239998 0.268099993 0.164450010

0.235929990 0.725990023 0.036670001

0.298300004 0.726000000 0.889599991

0.339949989 0.611519991 0.953490003

0.350419998 0.611519991 0.090920003

0.201909995 0.622850011 0.118390004

0.277579999 0.611519991 0.100560006

0.381610012 0.726000000 0.017379999

0.319239998 0.497049996 0.164450010

0.247230005 0.726989949 0.176139990

0.381610012 0.497049996 0.017379999

0.308769989 0.726000000 0.027030001

0.319239998 0.725990023 0.164450010

0.245169997 0.500599993 0.175620000

0.292430019 0.381019991 0.231779989

0.289330006 0.609749981 0.232710012

0.196066999 0.615760033 0.380449994

1. **Transition state structure in Figure 7a.**

1.0

40.0000000000 0.0000000000 0.0000000000

0.0000000000 12.8087997437 0.0000000000

0.0000000000 0.0000000000 30.0000000000

Cu O C

42 62 1

Direct

0.026629999 0.085330001 0.132049990

0.032269999 0.091270000 0.295799987

0.027430001 0.253259996 0.216719993

0.026910001 0.250710018 0.047260002

0.080669999 0.086230000 0.216760000

0.130669999 0.084850002 0.131259990

0.140540004 0.110279996 0.299549993

0.080390000 0.250860007 0.132119997

0.086369997 0.270310010 0.300229994

0.134169996 0.253259996 0.216719993

0.080150002 0.083619993 0.047200000

0.181500006 0.101839997 0.214849997

0.226859999 0.106890003 0.302029991

0.190320003 0.277660003 0.298390007

0.026629999 0.418660003 0.132049990

0.032269999 0.424599993 0.295799987

0.027430001 0.586589978 0.216719993

0.026910001 0.584049996 0.047260002

0.080669999 0.419559973 0.216760000

0.130669999 0.418179994 0.131259990

0.140540004 0.443619984 0.299549993

0.080390000 0.584190008 0.132119997

0.086369997 0.603640012 0.300229994

0.134169996 0.586589978 0.216719993

0.080150002 0.416950004 0.047200000

0.181500006 0.435169980 0.214849997

0.226859999 0.440220013 0.302029991

0.189219999 0.606800015 0.299969991

0.026629999 0.751990023 0.132049990

0.032269999 0.757930013 0.295799987

0.027430001 0.919930013 0.216719993

0.026910001 0.917379942 0.047260002

0.080669999 0.752889956 0.216760000

0.130669999 0.751510014 0.131259990

0.137279999 0.771419999 0.300850010

0.080390000 0.917519991 0.132119997

0.086369997 0.936980046 0.300229994

0.134169996 0.919930013 0.216719993

0.080150002 0.750290001 0.047200000

0.181500006 0.768499963 0.214849997

0.226859999 0.773549996 0.302029991

0.190320003 0.944320006 0.298390007

0.000830000 0.169970013 0.258869998

0.002190000 0.203299996 0.303409990

0.000530000 0.169450004 0.174810012

0.999930000 0.167600011 0.089390000

0.000230000 0.167140012 0.004840000

0.053920001 0.002820000 0.174339994

0.064570004 0.040199999 0.334549999

0.107570004 0.169980009 0.258869998

0.122529995 0.218370002 0.332979997

0.054189998 0.003200000 0.258890009

0.107270002 0.169450004 0.174810012

0.053679997 0.000090000 0.004790000

0.106669998 0.167600011 0.089390000

0.053429997 0.000810000 0.089960003

0.156169999 0.016180000 0.174310001

0.179809999 0.065959999 0.330270004

0.207410002 0.179989986 0.254850006

0.228630018 0.251399989 0.337130006

0.160929990 0.003200000 0.258890009

0.249919987 0.332530005 0.328999996

0.000830000 0.503310029 0.258869998

0.002190000 0.536629961 0.303409990

0.000530000 0.502780024 0.174810012

0.999930000 0.500930013 0.089390000

0.000230000 0.500469995 0.004840000

0.053920001 0.336149989 0.174339994

0.064570004 0.373530005 0.334549999

0.107570004 0.503310029 0.258869998

0.122529995 0.551709999 0.332979997

0.054189998 0.336530005 0.258890009

0.107270002 0.502780024 0.174810012

0.053679997 0.333429975 0.004790000

0.106669998 0.500930013 0.089390000

0.053429997 0.334140012 0.089960003

0.156169999 0.349510014 0.174310001

0.179809999 0.399290001 0.330270004

0.207410002 0.513320043 0.254850006

0.228780007 0.583339997 0.337919998

0.160929990 0.336530005 0.258890009

0.249919987 0.665870002 0.328999996

0.000830000 0.836640012 0.258869998

0.002190000 0.869960018 0.303409990

0.000530000 0.836109969 0.174810012

0.999930000 0.834259996 0.089390000

0.000230000 0.833809992 0.004840000

0.053920001 0.669489986 0.174339994

0.064570004 0.706860025 0.334549999

0.107570004 0.836640012 0.258869998

0.119920003 0.879839997 0.334950002

0.054189998 0.669870002 0.258890009

0.107270002 0.836109969 0.174810012

0.053679997 0.666760033 0.004790000

0.106669998 0.834259996 0.089390000

0.053429997 0.667480009 0.089960003

0.156169999 0.682849974 0.174310001

0.207410002 0.846649989 0.254850006

0.228630018 0.918069987 0.337130006

0.160929990 0.669870002 0.258890009

0.249919987 0.999199985 0.328999996

0.175865996 0.725167002 0.333182017

0.180135989 0.731946988 0.422183990

0.166477990 0.692060978 0.513535023

0.185353994 0.729438984 0.486833000

1. **Transition state structure in Figure 7b.**

1.0

40.0000000000 0.0000000000 0.0000000000

0.0000000000 12.8087997437 0.0000000000

0.0000000000 0.0000000000 30.0000000000

Cu O Ag C

42 62 30 1

Direct

0.026629999 0.085330001 0.252950017

0.030920002 0.088129998 0.417169984

0.027430001 0.253259996 0.337620004

0.026910001 0.250710018 0.168160009

0.080669999 0.086230000 0.337660027

0.127880001 0.077169999 0.251710002

0.136580002 0.096870002 0.421439997

0.080390000 0.250860007 0.253019985

0.083849996 0.262279987 0.422019990

0.134169996 0.253259996 0.337620004

0.075520003 0.082570006 0.166200002

0.181900001 0.097730000 0.332030010

0.244689989 0.138699994 0.408570004

0.189759994 0.270139993 0.416640027

0.026629999 0.418660003 0.252950017

0.031009999 0.421400007 0.417090003

0.027430001 0.586589978 0.337620004

0.026910001 0.584049996 0.168160009

0.080669999 0.419559973 0.337660027

0.132660007 0.422569987 0.249319998

0.137520003 0.430209999 0.421119976

0.080390000 0.584190008 0.253019985

0.084259999 0.596010014 0.422139994

0.134169996 0.586589978 0.337620004

0.075819999 0.415640012 0.166370010

0.186609995 0.435149989 0.336200015

0.239860010 0.479290001 0.427549998

0.188300002 0.610319969 0.422159990

0.026629999 0.751990023 0.252950017

0.031029999 0.754469958 0.417079989

0.027430001 0.919930013 0.337620004

0.026910001 0.917379942 0.168160009

0.080669999 0.752889956 0.337660027

0.130299997 0.746949966 0.250200001

0.135640001 0.770849974 0.422760010

0.080390000 0.917519991 0.253019985

0.083679998 0.930190008 0.422010008

0.134169996 0.919930013 0.337620004

0.075790000 0.748640012 0.166229995

0.186479998 0.757690045 0.333050028

0.237809992 0.783789964 0.419250011

0.187709999 0.933690045 0.419090017

0.000830000 0.169970013 0.379770025

0.000940000 0.200870002 0.424689992

0.000530000 0.169450004 0.295709991

0.999930000 0.167600011 0.210290003

0.000230000 0.167140012 0.125740004

0.053920001 0.002820000 0.295240021

0.060460001 0.027699999 0.457219982

0.107570004 0.169970013 0.379770025

0.114830005 0.193500000 0.457649994

0.054189998 0.003200000 0.379790020

0.107270002 0.169450004 0.295709991

0.053679997 0.000090000 0.125689999

0.101460004 0.152129988 0.209330002

0.053429997 0.000810000 0.210859998

0.155799997 0.011649999 0.293450006

0.167089999 0.028010000 0.457100010

0.209940004 0.184609988 0.366639996

0.219390011 0.201040001 0.450070000

0.160929990 0.003200000 0.379790020

0.282019997 0.077040001 0.405349986

0.000830000 0.503310029 0.379770025

0.001050000 0.534099993 0.424710019

0.000530000 0.502780024 0.295709991

0.999930000 0.500930013 0.210290003

0.000230000 0.500469995 0.125740004

0.053920001 0.336149989 0.295240021

0.060610002 0.360790001 0.457060019

0.107570004 0.503310029 0.379770025

0.116899991 0.530439990 0.456739998

0.054189998 0.336530005 0.379790020

0.107270002 0.502780024 0.295709991

0.053679997 0.333429975 0.125689999

0.101880002 0.487919999 0.208840005

0.053429997 0.334140012 0.210859998

0.162129998 0.356720013 0.290469996

0.169729996 0.362800015 0.456010024

0.209590006 0.518939990 0.378549989

0.217529988 0.565989986 0.462330023

0.160929990 0.336530005 0.379790020

0.270079994 0.387939990 0.412519995

0.000830000 0.836640012 0.379770025

0.001100000 0.867359988 0.424750010

0.000530000 0.836109969 0.295709991

0.999930000 0.834259996 0.210290003

0.000230000 0.833809992 0.125740004

0.053920001 0.669489986 0.295240021

0.060839999 0.694440027 0.457050006

0.107570004 0.836640012 0.379770025

0.114390004 0.867459981 0.458349991

0.054189998 0.669870002 0.379790020

0.107270002 0.836109969 0.295709991

0.053679997 0.666760033 0.125689999

0.101539993 0.818860025 0.209439993

0.053429997 0.667480009 0.210859998

0.160160005 0.676930013 0.290419992

0.209780002 0.842900007 0.373049990

0.215210009 0.865510014 0.456300004

0.160929990 0.669870002 0.379790020

0.268830013 0.696139975 0.403330008

0.170140994 0.715744025 0.461706003

0.170732999 0.709041024 0.504365031

0.185000002 0.796781029 0.620543035

0.267109990 0.382569987 0.084039998

0.267109990 0.611519991 0.084039998

0.298300004 0.497049996 0.010500000

0.235929990 0.268099993 0.157570012

0.298300004 0.268099993 0.010500000

0.235929990 0.497049996 0.157570012

0.277579999 0.382569987 0.221459993

0.339949989 0.382569987 0.074389998

0.350419998 0.382569987 0.211819998

0.204279995 0.387199985 0.236300008

0.381610012 0.268099993 0.138280010

0.308769989 0.497049996 0.147930002

0.234980011 0.287339978 0.314660009

0.308769989 0.268099993 0.147930002

0.319239998 0.268099993 0.285350005

0.235929990 0.725990023 0.157570012

0.298300004 0.726000000 0.010500000

0.339949989 0.611519991 0.074389998

0.350419998 0.611519991 0.211819998

0.201909995 0.622850011 0.239289999

0.277579999 0.611519991 0.221459993

0.381610012 0.726000000 0.138280010

0.319239998 0.497049996 0.285350005

0.247230005 0.726989949 0.297039986

0.381610012 0.497049996 0.138280010

0.308769989 0.726000000 0.147930002

0.319239998 0.725990023 0.285350005

0.245169997 0.500599993 0.296519979

0.292430019 0.381019991 0.352679984

0.289330006 0.609749981 0.353610007

0.189551997 0.794303030 0.583322016

**REFERENCES**

S1. Michaelides, A., Reuter, K. & Scheffler, M. When seeing is not believing: Oxygen on Ag (111), a simple adsorption system? *J. Vac. Sci. Technol. A* **23**, 1487-1497 (2005).
